# Downregulation of CHIP promotes ovarian cancer metastasis by inducing Snail‐mediated epithelial–mesenchymal transition

**DOI:** 10.1002/1878-0261.12485

**Published:** 2019-04-08

**Authors:** Sun‐Mi Park, Seung‐Ho Park, Ki‐Jun Ryu, In‐Kyu Kim, Hyeontak Han, Hyo‐Jin Kim, Seon‐Hee Kim, Keun‐Seok Hong, Hyemin Kim, Minju Kim, Bok Im Cho, Jeong Doo Heo, Na Hyun Kim, Eun Mi Hwang, Jae‐Yong Park, Jong In Yook, Hee Jun Cho, Cheol Hwangbo, Kwang Dong Kim, Hoseok Song, Jiyun Yoo

**Affiliations:** ^1^ Division of Applied Life Science (BK21 Plus) Research Institute of Life Sciences Gyeongsang National University Jinju Korea; ^2^ Environmental Disease Research Center Korea Research Institute of Bioscience and Biotechnology Daejeon Korea; ^3^ Gyeongnam Department of Environmental Toxicology and Chemistry Toxicology Screening Center Korea Institute of Toxicology Jinju Korea; ^4^ Center for Functional Connectomics Korea Institute of Science and Technology Seoul Korea; ^5^ School of Biosystem and Biomedical Science College of Health Science Korea University Seoul Korea; ^6^ Department of Oral Pathology Oral Cancer Research Institute College of Dentistry Yonsei University Seoul Korea; ^7^ Immunotherapy Convergence Research Center Korea Research Institute of Bioscience and Biotechnology Daejeon Korea; ^8^ Division of Life Science Gyeongsang National University Jinju Korea; ^9^ Department of Biomedical Sciences College of Medicine Korea University Seoul Korea

**Keywords:** cancer metastasis, CHIP, E3 ubiquitin ligase, EMT, ovarian cancer, snail

## Abstract

The epithelial–mesenchymal transition (EMT) plays a pivotal role in the conversion of early‐stage tumors into invasive malignancies. The transcription factor Snail, an extremely unstable protein whose subcellular levels are regulated by many E3 ubiquitin ligases, promotes EMT as well as associated pathological characteristics including migration, invasion, and metastasis. Through yeast two‐hybrid screening, we identified the carboxyl terminus of Hsc70‐interacting protein (CHIP) as a novel Snail ubiquitin ligase that interacts with Snail to induce ubiquitin‐mediated proteasomal degradation. Inhibition of CHIP expression increases Snail protein levels, induces EMT, and enhances *in vitro* migration and invasion as well as *in vivo* metastasis of ovarian cancer cells. In turn, Snail depletion abrogates all phenomena induced by CHIP depletion. Finally, Snail and CHIP expression is inversely correlated in ovarian tumor tissues. These findings establish the CHIP–Snail axis as a post‐translational mechanism of EMT and cancer metastasis regulation.

**Abbreviations**
AD: activation domain
BD: binding domain
CHIP: carboxyl terminus of Hsc70‐interacting protein
CHX: cycloheximide
EMT: epithelial–mesenchymal transition
GSK‐3β: glycogen synthase kinase‐3β
HDAC: histone deacetylase
NES: nuclear export sequence
SD: standard deviation
shRNA: short hairpin RNA
SRD: Ser‐rich domain
Ub: ubiquitin

## 1 Introduction

The majority of cancer deaths are attributable to the local invasion and distant metastasis of tumor cells (Wan *et al*., 2013). During metastasis, epithelial cells initially lose apical–basal polarity and cell–cell contact while shifting to a mesenchymal phenotype (Ikenouchi *et al*., 2003). This loss of epithelial features is often accompanied by increased cell motility and expression of mesenchymal genes, a process that is collectively referred to as the epithelial–mesenchymal transition (EMT) and considered a key step during the progression of tumors toward metastasis (Savanger, 2001; Thiery *et al*., 2009). Thus, EMT regulators, which likely play important roles in cancer progression, have been extensively studied. One such regulator is the zinc finger protein Snail, which induces EMT via the direct repression of E‐cadherin transcription during development or tumor progression. Accordingly, the loss of expression of E‐cadherin, a central component of cell–cell adhesion junctions required for embryonic epithelial formation and epithelial homeostasis in adults, is a hallmark of EMT (Ikenouchi *et al*., 2003). The association of a loss of E‐cadherin expression in tumors with poor clinical outcomes has led to the use of E‐cadherin repressors as markers of malignancy (Nieto, 2002; Thiery, 2002).

Snail is an extremely unstable protein, and its subcellular levels are mainly regulated by the E3 ubiquitin ligases FBXW1 (β‐TrCP1), SPSB3, FBW7, FBXL14, FBXL5, and FBXO11 (Liu *et al*., 2018; Vinas‐Castells *et al*., 2010, 2014; Zhang *et al*., 2018; Zheng *et al*., 2014; Zhou *et al*., 2004). Snail undergoes proteasomal degradation after glycogen synthase kinase‐3β (GSK‐3β)‐dependent phosphorylation and β‐TrCP1‐mediated ubiquitylation (Zhou *et al*., 2004). Two GSK‐3β consensus sites are located on Snail. At the first of these, in the nucleus, GSK‐3β phosphorylates four target serine residues adjacent to a nuclear export sequence (NES), leading to unmasking of the NES and subsequent export of Snail from the nucleus (Dominguez *et al*., 2003). At the second site, in the cytosol, GSK‐3β phosphorylates the remaining two serine residues, which overlap with a destruction motif recognized by the E3 ubiquitin ligase β‐TrCP1. In turn, phosphorylated Snail binds to β‐TrCP1 and is subsequently ubiquitylated and degraded (Zhou *et al*., 2004). Recent studies have suggested that GSK‐3β‐dependent phosphorylation of Snail is also crucial for SPSB3‐ and FBW7‐mediated Snail ubiquitylation and degradation (Liu *et al*., 2018; Zhang *et al*., 2018). Activation of Akt kinase or Wnt signaling inhibits Snail phosphorylation by inactivating GSK‐3β, resulting in increased Snail protein levels (Yook *et al*., 2005). However, GSK‐3β inactivation does not always increase the stability of Snail because another E3 ubiquitin ligase, FBXL14, also interacts with it and promotes its ubiquitylation and proteasomal degradation independently of GSK‐3β‐mediated phosphorylation (Vinas‐Castells *et al*., 2010). Under hypoxic conditions, Snail is stabilized concomitantly with strong downregulation of FBXL14 mRNA expression. FBXL5 was identified as a novel Snail ubiquitin ligase via short hairpin RNA screening (Vinas‐Castells *et al*., 2014). FBXL5 is located in the nucleus, where it promotes Snail ubiquitylation and affects Snail protein stability and function by impairing DNA binding. However, FBXL5 is highly sensitive to stress, and its expression is downregulated by iron depletion and γ‐irradiation, a finding that explains the stabilization of Snail under these conditions. A recent luciferase‐based, genome‐wide E3 ligase siRNA library screening protocol identified FBXO11 as another important E3 ubiquitin ligase that targets Snail for ubiquitylation and degradation (Zheng *et al*., 2014). FBXO11‐mediated regulation is dependent on the phosphorylation of Snail Serine‐11 by PKD1. On the other hand, Pellino‐1, a RING‐like domain containing E3 ubiquitin ligase, has been known to enhance Snail stability through Lys(K)63‐mediated polyubiquitination which contributes to lung tumorigenesis by promoting EMT (Jeon *et al*., 2017).

The carboxyl terminus of Hsc70‐interacting protein (CHIP), also known as Stub1, was originally identified as a U‐box‐type E3 ubiquitin ligase and co‐chaperone of Hsc70 (Ballinger *et al*., 1999). CHIP functions as a tumor suppressor by catalyzing the ubiquitylation and degradation of several oncogenic proteins, including ErbB2 (Xu *et al*., 2002), estrogen receptor α (Tateishi *et al*., 2004), Met receptor (Jang *et al*., 2011), c‐Myc (Paul *et al*., 2013), NF‐κB (Wang *et al*., 2013), and Slug (Kao *et al*., 2014). Recently, CHIP was reported to play a critical role in breast cancer metastasis by acting as an upstream regulator of oncogenic pathways and suppressing tumor progression through the degradation of oncogenic proteins, including SRC‐3 (Kajiro *et al*., 2009). However, the relationship between CHIP and tumor progression has not been fully elucidated.

In this study, we used a yeast two‐hybrid screening to identify CHIP as a new U‐box‐type E3 ubiquitin ligase that targets Snail for ubiquitylation and degradation. We further demonstrate that CHIP controls the stability of Snail protein, as well as Snail‐mediated EMT, migration, and invasion and the metastatic potential of ovarian cancer cells.

## 2 Materials and methods

### 2.1 Cell culture and reagents

All cell lines used in this study were obtained from the Korean Cell Line Bank (Seoul, Korea), where they were characterized by DNA fingerprinting and isozyme detection, and cultured according to American Type Culture Collection instructions. Human HEK293T, human colon cancer cell lines (HCT‐116, SW480, SW620, HT‐29, LoVo), and human breast cancer cell lines (MCF7, MDA‐MB‐231) were cultured in DMEM (Invitrogen, Waltham, MA, USA) supplemented with 10% FBS and 1% penicillin and streptomycin. Human ovarian cancer cell lines (SKOV3, OVCAR3, SNU‐8, SNU‐119, SNU‐251) were cultured in RPMI (Invitrogen) supplemented with 10% FBS and 1% penicillin and streptomycin. All plasmids were transfected into different cell lines using FuGENE 6 following the manufacturer's manual (Roche, Basel, Switzerland). The short hairpin RNA (shRNA)‐expressing lentiviral transduction particles for targeting the CHIP and Snail genes were constructed by inserting synthetic double‐stranded oligonucleotides (Table S1) into the pLKO.1 lentiviral vector, and the Non‐Target shRNA Control Transduction Particles were purchased from Sigma (St. Louis, MO, USA). Forty‐eight hours after transduction, 1 μg·mL^−1^ puromycin (Takara, Kyoto, Japan) was added to the cultures for selection. After 14 days, puromycin‐resistance cell pools were established. Proteasome inhibitor (MG132) and cycloheximide (CHX) were purchased from Calbiochem (Burlington, MA, USA).

### 2.2 Yeast two‐hybrid screening

The Snail gene was cloned into pGBK7, which encodes a GAL4 DNA‐binding domain (BD), while the CHIP gene was cloned into pGADT7, which encodes an activation domain (AD). To evaluate interactions of the two proteins, both BD‐Snail and AD‐CHIP were cotransformed into the yeast strain AH109. The transformed yeast was then cultured in synthetic dropout (SD) medium lacking Leu, Trp, and His (LTH‐) for growth selection.

### 2.3 Gene cloning and transfection

The full‐length human CHIP gene was subcloned into a pDONR207 vector (Entry vector) using the Gateway Cloning System (Invitrogen) following the manufacturer's instructions. The entry clones were converted into several destination vectors, pDEST‐GFP‐C, pDEST‐FLAG‐C, and pDEST‐HA‐C. For transient transfection, HEK293T cells were seeded in 6‐well or 100‐mm‐diameter dish for 24 h and transfected with the indicated plasmid by using FuGENE 6 transfection reagent (Roche) following the manufacturer's instructions. After 48 h, the cells were harvested and used for western blot analysis.

### 2.4 Western blot analyses

Triton X‐100 lysis buffer [20 mm Tris (pH 7.4), 2 mm EDTA, 150 mm sodium chloride, 1 mm sodium deoxycholate, 1% Triton X‐100, 10% glycerol, 2 pills protease inhibitor cocktail (Roche)] was used to collect protein from cells. Protein samples were heat‐denatured and equally loaded, separated on 8–12% SDS/PAGE gel, transferred onto a polyvinylidene difluoride membrane (GE Healthcare, Buckinghamshire, UK), and blocked with 5% nonfat dry milk. Primary antibodies for western blot analyses included mouse anti‐α‐tubulin (1 :  20 000 dilution; Sigma), rabbit anti‐Snail (1 : 1000 dilution; Cell Signaling, Danvers, MA, USA), rabbit anti‐Slug (1 : 1000 dilution; Cell Signaling), rabbit anti‐CHIP (1 : 1000 dilution; Abgent, San Diego, CA, USA), mouse anti‐Flag (1 : 3000 dilution; Abcam), mouse anti‐GFP (1 : 1000 dilution; Santa Cruz), mouse anti‐HA (1 : 1000 dilution; Abcam, Cambridge, MA, USA), mouse anti‐Vimentin (1 : 1000 dilution; Santa Cruz, Santa Cruz, CA, USA), mouse anti‐E‐cadherin (1 : 5000 dilution; BD Biosciences, San Jose, CA, USA), and mouse anti‐Ub (1 : 5000 dilution; Santa Cruz). Membranes were incubated with horseradish peroxidase‐conjugated anti‐mouse or anti‐rabbit secondary antibody (1 : 5000 dilution; Cell Signaling) for 1 h, and chemiluminescence signals were detected by ECL substrate (GE Healthcare, Chicago, IL, USA).

### 2.5 Total RNA extraction and RT‐PCR

Total RNA was extracted from the cultured cells using RNeasy Mini Kit (Qiagen, Hilden, Germany) following the manufacturer's instructions. RT‐PCR was performed using AccuPower RT‐PCR PreMix kit (Bioneer, Daejeon, South Korea) according to the manufacturer's instructions. Amplification was performed using Thermo Electron PCR thermal cycler. Primers used were Snail (F: 5′‐TGCAGTATTTGCAGTTGAAG‐3′; R: 5′‐CAGAGTTTACCTTCCAGCAG‐3′), CHIP (F: 5′‐CGACTACCTGTGTGGCAAGA‐3′; R: 5′‐CAAGTTGGGGATGAGCTGTT‐3′), and β‐actin (F: 5′‐GTGGGGCGCCCCAGGCACCA‐3′; R: 5′‐CTCCTTAATGTCACGCACGA‐3′).

### 2.6 Co‐immunoprecipitation assay

The Flag‐CHIP (wild‐type or truncated mutants) plasmids were cotransfected with HA‐tagged Snail plasmid into HEK293T cells in 10‐cm plates using FuGENE 6 transfection reagent (Roche) following the manufacturer's instructions. Two days after transfection, the cells were treated with 10 μm MG132 for 6 h. One milligram cell lysates was then collected and immunoprecipitated with either 2 μg Flag or HA antibody together with 30 μL protein A/G beads (Santa Cruz) at 4 °C overnight. After extensive washing (4 times), the beads were spun down and resuspended with 30 μL 1 × SDS buffer. After boiling for 5 min, pull‐down samples were run on SDS/PAGE gel along with 5% input sample and transferred to PVDF membrane to be detected with appropriate antibodies as described in each figure.

### 2.7 Ubiquitylation assay

Ubiquitylation assay was done following a co‐IP protocol. HEK293T cells were transfected with HA‐Ub, GFP‐Snail, and CHIP wild‐type or mutants. Two days after transfection, cells were treated with 10 μm MG132 for 12 h to block proteasomal degradation of the Snail protein before lysing with Triton X‐100 lysis buffer. One milligram cell lysates was then collected and immunoprecipitated with anti‐GFP antibody together with 30 μL protein A/G beads to specifically pull down GFP‐Snail protein. Pulled‐down samples were subject to immunoblotting with anti‐HA (Ubiquitin) to visualize polyubiquitylated Snail protein bands. For ubiquitylation assay under denaturing conditions, denaturing immunoprecipitation was performed. HEK293T cells transfected with HA‐Ub, GFP‐Snail, and CHIP wild‐type or mutants were treated with 10 μm MG132 for 12 h before harvest. Cells were lysed with denaturing lysis buffer [50 mm Tris (pH 7.4), 5 mm MgCl_2_, 150 mm potassium chloride, 1% Triton X‐100, 5% glycerol, 2 mm β‐mercaptoethanol, 2 pills protease inhibitor cocktail (Roche)], which was used to collect protein from cells. About 1% SDS was added to the cell lysates, which were then boiled for 5 min, and diluted to yield 0.1% SDS final concentration using the denaturing lysis buffer. The denatured lysates were immunoprecipitated with anti‐GFP antibody and subjected to immunoblot analysis.

### 2.8 Migration and invasion assays

For wound‐healing assays, 4.0 × 10^4^ cells in 70 μL of medium were seeded into Culture‐Insert (Ibidi, Munich, Germany). After the cells were confluent, to inhibit the effect of cell proliferation, the cells were pretreated with 10 μg·mL^−1^ mitomycin C (Sigma) for 2 h and washed with culture medium. After removal of Culture‐Insert, cells were incubated with fresh media and photographs of the migration assay were taken at 0 and 16 h using a phase‐contrast microscope with digital camera.

Invasion assays were performed using QCM™ 24‐Well Cell Invasion Assay kit (Chemicon, Burlington, MA, USA) following the manufacturer's instructions. A total of 2.5 × 10^5^ cells in 250 μL of medium were placed in the insert and allowed to invade for 20 h. The lower chamber was filled with 500 μL of appropriated media containing 20% FBS. After incubation, cells/medium remaining on the top side of insert was removed by pipetting. The invasion chamber insert was transferred into a clean well containing 225 μL of prewarmed Cell Detachment Solution and incubated for 30 min at 37 °C. The insert was removed from the well. About 75 μL of Lysis Buffer/Dye solution (CyQUANT GR Dye 1 : 75 with 4 × Lysis Buffer) was added into each well and incubated for 15 min at room temperature. Two hundred microliters of the mixture was transferred into a 96‐well plate and assessed with a fluorescence plate reader using a 480/520‐nm filter set.

### 2.9 Animal studies

All animal experiments were approved by the Institutional Animal Care and Use Committees (IACUC) of Korea Institute of Toxicology Gyeongnam Department of Environmental Toxicology and Chemistry and followed National Research Council Guidelines. A total of 2 × 10^6^ of SKOV3 (shCont), CHIP‐depleted SKOV3 (shCHIP‐1), or Snail and CHIP double‐depleted SKOV3 (shCHIP‐1/shSnail‐2) cells were injected intraperitoneally into female BALB/c nude mice (*n* = 15 for each group). Six weeks after the injection, the mice were euthanized, and primary tumor masses in the peritoneum for ovarian cancer and lung were fixed in 4% paraformaldehyde for 24 h. Sections (4 μm) were stained with hematoxylin and eosin. The number of tumor masses in the lung was counted under a dissecting microscope.

### 2.10 Immunohistochemistry

Formalin‐fixed paraffin‐embedded microarrays of de‐identified ovarian cancer tissues (28 cases) and normal ovarian tissues were from ISU Abxis (Seongnam, South Korea). Twenty‐two cases of ovarian cancer tissue specimens were collected from 2001 to 2012 at Gyeongsang National University Hospital, Jinju, Korea. These clinical ovarian cancer tissue specimens were examined and diagnosed by pathologists at Gyeongsang National University Hospital. Tumor collections with informed consents and analyses were approved by the Institutional Review Board (IRB) at Gyeongsang National University Hospital, Jinju, Korea. The staining score was determined by two pathologists blinded to the tumor information using the method described below. ISU Abxis AccuMax TMA samples (A213III) were stained at histology core facility at ISU Abxis. Immunoreactivity was scored as positive when 50% or more cancer cells showed reactivity. The positive samples were classified further into weak positive and positive based on their intensity of reactivity. The staining intensity was also scored on a three‐tiered scale (negative, weak positive, and positive). Primary antibodies used in the staining were anti‐Snail (1 : 50; Cell Signaling) and anti‐CHIP (1 : 100; Abgent).

### 2.11 Statistical analysis

Statistical analysis was performed using the pasw statistics 18.0 software (IBM Corporation, Somers, NY, USA). Data represent the mean ± SD. The significance of the differences was determined using the chi‐square test. The relationships between CHIP and Snail expression were analyzed by Fisher's exact test. Statistical analysis was performed using *t*‐test. *P* values < 0.05 were considered to be statistically significant.

## 3 Results

### 3.1 Identification of CHIP that interacts with Snail

**Figure 1:**
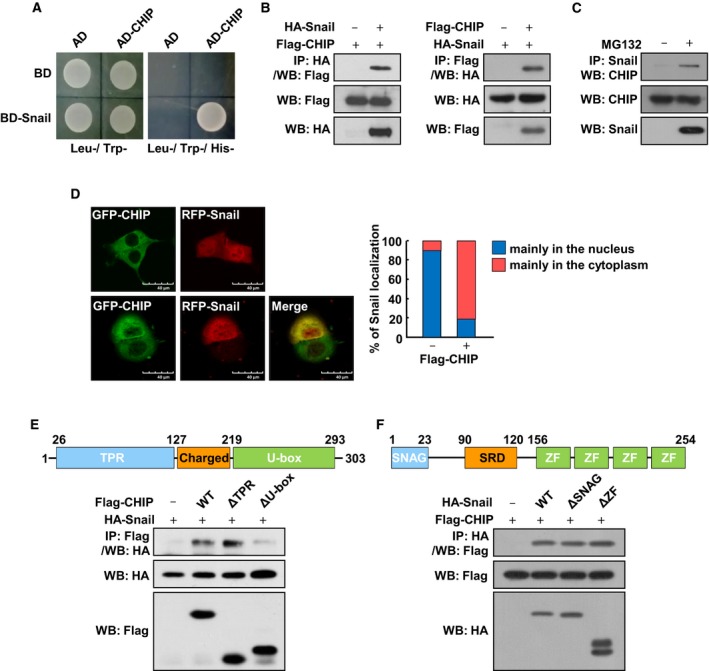
CHIP interacts with Snail. (A) Yeast two‐hybrid assays of interactions between CHIP and Snail. (B) Interaction between exogenous Snail and CHIP. HA‐Snail and Flag‐CHIP were expressed in HEK293T cells and treated with 10 μm MG132 for 6 h before harvest. Cell lysates were immunoprecipitated with an anti‐HA or anti‐Flag and analyzed by western blot using anti‐Flag (left) or anti‐HA (right) antibody, respectively. (C) Interaction between endogenous Snail and CHIP. SKOV3 cells were treated with 10 μm MG132 for 6 h before harvest. Cell lysates were immunoprecipitated with an anti‐Snail and analyzed by western blot using anti‐CHIP antibody. (D) Subcellular localization of CHIP and Snail. HEK293T cells were transfected with GFP‐CHIP and RFP‐Snail, respectively (upper), or cotransfected with GFP‐CHIP and RFP‐Snail (lower) and treated with 10 μm MG132 for 6 h before fixation. Cells were fixed and examined by confocal microscopy. Superimposing the two colors (merge) resulted in a yellow signal, indicating colocalization of the two proteins. Scale bars, 40 μm. The right panel presents the quantified value of the intracellular localization of Snail examined by confocal microscopy. (E) Interaction between Snail and CHIP deletion mutants. Lysates of HEK293T cells transfected with HA‐tagged Snail and Flag‐tagged fragments of CHIP subjected to immunoprecipitation with anti‐Flag antibody, followed by western blot with anti‐HA antibody. (F) Interaction between CHIP and Snail deletion mutants. Lysates of HEK293T cells transfected with Flag‐tagged CHIP and HA‐tagged fragments of Snail subjected to immunoprecipitation with anti‐HA antibody, followed by western blot with anti‐Flag antibody.

To identify novel Snail‐interacting proteins that could regulate Snail function, we performed a yeast two‐hybrid screening with full gene of Snail as a bait. Among the positive clones independently isolated from the HeLa cell cDNA library, we focused particularly on CHIP because this protein has been identified as a tumor suppressor that can induce the ubiquitylation and degradation of several oncogenic proteins (Jang *et al*., 2011; Kajiro *et al*., 2009; Kao *et al*., 2014; Paul *et al*., 2013; Tateishi *et al*., 2004; Wang *et al*., 2013; Xu *et al*., 2002). The protein–protein interaction between CHIP and Snail was confirmed using the yeast two‐hybrid assays (Fig. 1A). To investigate the interaction between CHIP and Snail *in vivo*, N‐terminal tagged vectors expressing HA‐tagged Snail (HA‐Snail) and Flag‐tagged CHIP (Flag‐CHIP) were cotransfected into HEK293T cells and treated with the proteasome inhibitor MG132. Immunoprecipitation with an anti‐HA antibody, followed by western blotting with an anti‐Flag antibody, showed that Flag‐CHIP interacts with HA‐Snail (Fig. 1B, left), and *vice versa* (Fig. 1B, right). Next, we confirmed the interaction between endogenous Snail and CHIP proteins via co‐IP experiments using MG132‐treated SKOV3 ovarian cancer cells (Fig. 1C). We next checked the subcellular localization of CHIP and Snail. When CHIP and Snail were expressed, respectively, CHIP was found only in the cytoplasm and Snail was mainly localized to the nucleus, with a weak signal in the cytoplasm (Fig. 1D, upper and right). We also found that when CHIP was co‐expressed with Snail, these proteins were colocalized mainly in the cytoplasm (Fig. 1D, lower and right). To determine which CHIP motif is required for interaction with Snail, we co‐expressed two truncated forms of CHIP, CHIP‐ΔTPR, in which the Hsp‐binding TPR domain was deleted, and CHIP‐ΔU‐box, in which the U‐box domain required for ubiquitylation was deleted (Ballinger *et al*., 1999), along with full‐length Snail and subjected the resulting cells to IP using an anti‐Flag antibody. In this experiment, the U‐box domain, but not the TPR domain, retained the ability to associate with Snail (Fig. 1E). Based on these data, we concluded that Snail interacts with the U‐box domain of CHIP. We also found that the two truncated forms of Snail, Snail‐ΔSNAG and Snail‐ΔZF, with the HDAC‐BD and the DNA‐BD deleted, respectively, retain the ability to associate with CHIP to the same extent as Snail‐WT (Fig. 1F). These results suggest that the region containing Ser‐rich domain (SRD) might be a CHIP‐interacting domain in Snail.

### 3.2 CHIP downregulates Snail protein level by enhancing its ubiquitylation‐dependent degradation

**Figure 2:**
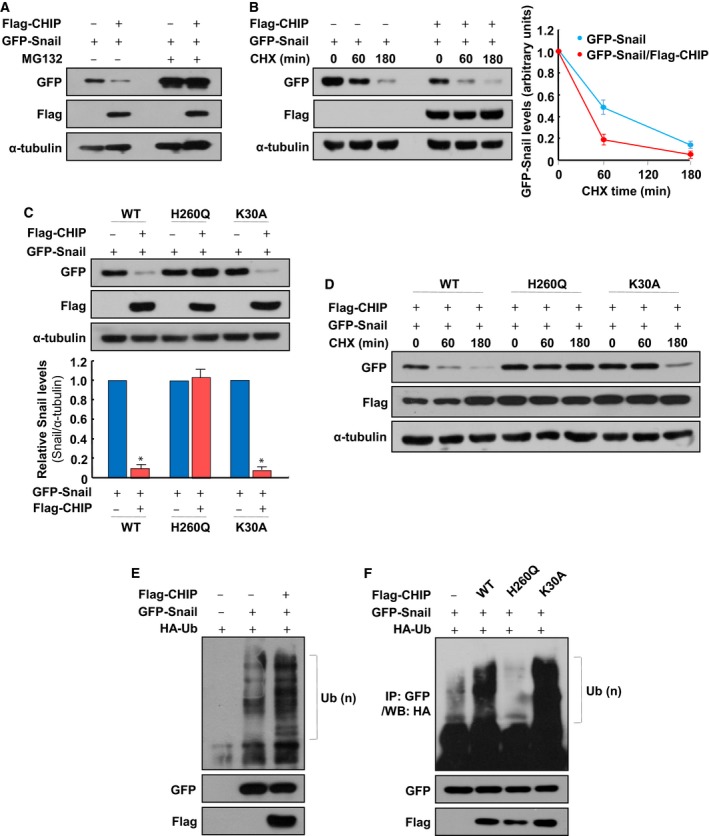
CHIP ubiquitylates Snail in a U‐box‐dependent manner. (A) Degradation of Snail by CHIP. HEK293T cells were transfected with GFP‐Snail and Flag‐CHIP and treated with 10 μm MG132 for 6 h before harvest, and western blot was performed with GFP‐ and Flag‐specific antibodies. (B) Destabilization of Snail by CHIP. HEK293T cells were transfected with plasmids expressing GFP‐Snail and Flag‐CHIP and treated with 20 μg·mL^−1^ CHX for the indicated times before harvest, and western blot was performed with GFP‐ and Flag‐specific antibodies. The right panel presents the mean ± SD of the densitometric analyses of GFP‐Snail levels in three independent experiments. (C) Effects of CHIP mutants on Snail degradation. GFP‐Snail was transfected into HEK293T cells with Flag‐CHIP, Flag‐CHIP‐H260Q, and Flag‐CHIP‐K30A, respectively. Cell lysates were subjected to western blot analysis using GFP‐ and Flag‐specific antibodies (upper). The data are representative of three independent experiments, and relative Snail levels were quantified using IMAGE J (NIH, Bethesda, MD, USA) software (lower). For normalization, α‐tubulin expression was used as a control. Data are mean ± SD of three independent experiments. *P <* *0.01 as determined by paired Student's *t*‐test. (D) Effects of CHIP mutants on Snail stability. GFP‐Snail was transfected into HEK293T cells with Flag‐CHIP, Flag‐CHIP‐H260Q, and Flag‐CHIP‐K30A, respectively. Each cell was treated with 20 μg·mL^−1^ CHX for the indicated times before harvest, and western blot was performed with GFP‐ and Flag‐specific antibodies. (E) CHIP‐dependent ubiquitylation of Snail. HEK293T cells were transfected with expression plasmids encoding GFP‐Snail, Flag‐CHIP, and HA‐ubiquitin and treated with 10 μm MG132 for 12 h before harvest. Lysates were immunoprecipitated using anti‐GFP antibody, followed by western blot with anti‐HA antibody (upper). Whole‐cell lysates were immunoblotted with anti‐GFP (middle) and anti‐Flag antibody (bottom) to demonstrate Snail and CHIP expression. Brackets indicate ubiquitylated Snail. (F) Ubiquitylation of Snail by CHIP WT, H260Q, and K30A mutants. Ubiquitylation assays using HEK293T cells transfected with the indicated plasmids were performed as in (E).

Based on the association of CHIP with Snail, we reasoned that CHIP might be an ubiquitin ligase for Snail degradation. Thus, to determine the effect of CHIP on Snail stability, we cotransfected HEK293T cells with GFP‐Snail and Flag‐CHIP genes. While CHIP expression strongly reduced the steady‐state Snail level, in the presence of MG132, CHIP‐induced Snail degradation was completely inhibited (Fig. 2A). In a CHX pulse‐chase analysis of GFP‐Snail expression, CHIP accelerated the degradation of this fusion protein (Fig. 2B). These results suggest that CHIP negatively regulates the stability of Snail through inducing its proteasomal degradation. Next, we found that CHIP‐K30A, a mutant incapable of binding to Hsp, but not CHIP‐H260Q, which lacks ubiquitin ligase activity, led to a significant reduction in Snail protein expression to the same levels as wild‐type CHIP did (Fig. 2C). In a CHX pulse‐chase analysis of GFP‐Snail expression, CHIP‐K30A, but not CHIP‐H260Q, accelerated the degradation of this fusion protein to the same levels as wild‐type CHIP did (Fig. 2D). These results suggest that CHIP mediates the degradation of Snail through its U‐box domain, thereby maintaining Snail protein at a low basal level, and that this effect is unrelated to the chaperone activity of CHIP. The U‐box dependence indicates that CHIP downregulates Snail via an ubiquitin‐mediated pathway. Accordingly, we conducted *in vivo* ubiquitylation experiments in HEK293T cells engineered to transiently overexpress GFP‐Snail and HA‐ubiquitin (Ub) with, or without, Flag‐CHIP and found that CHIP enhanced the ubiquitylation of Snail in the presence of MG132 (Fig. 2E). We also found that CHIP‐K30A, but not CHIP‐H260Q, could enhance the ubiquitylation of Snail similar to wild‐type CHIP (Fig. 2F). We have also shown that wild‐type CHIP and CHIP‐K30A could enhance the ubiquitylation of Snail under denaturing conditions, but CHIP‐H260Q could not (Fig. S1). All of these results suggest that CHIP acts as a direct E3 ubiquitin ligase on Snail and thereby induces Snail ubiquitylation and degradation.

Glycogen synthase kinase‐3β‐mediated phosphorylation is required for the ubiquitylation of Snail by the E3 ubiquitin ligase β‐TrCP (Zhou *et al*., 2004). To examine whether such phosphorylation is also required for CHIP‐mediated Snail degradation, we monitored steady‐state expression levels of the Snail‐6SA mutant, in which the six serine residues of the serine‐rich domain (including those phosphorylated by GSK‐3β) were mutated to alanine residues in the presence of CHIP. Interestingly, in the presence of ectopically expressed CHIP, the steady‐state levels of Snail‐6SA were reduced to a level comparable to Snail‐WT (Fig. S2A), and Snail‐6SA ubiquitylation was observed (Fig. S2B). These results indicate that CHIP can ubiquitylate and degrade Snail in a GSK‐3β‐independent manner.

### 3.3 Depletion of CHIP expression induces EMT and increases the migration and invasion abilities of ovarian cancer cells

**Figure 3:**
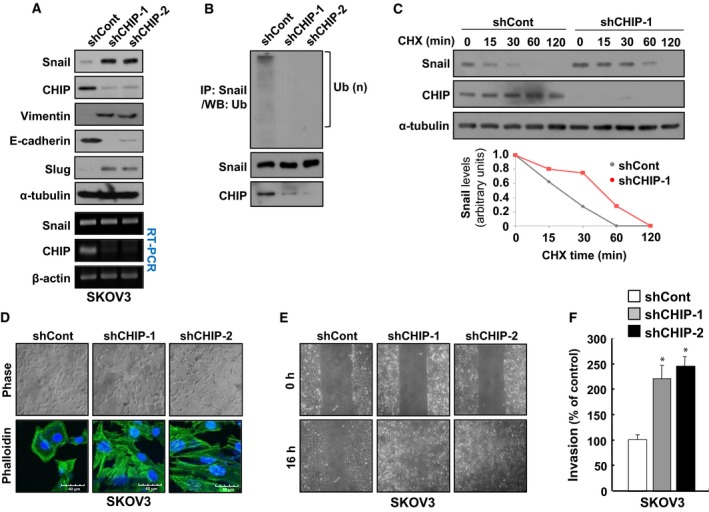
Depletion of CHIP expression induces EMT and increases the migration and invasion abilities of ovarian cancer cells. (A) Inhibition of CHIP induces changes of EMT markers. Lysates of SKOV3/shCont, SKOV3/shCHIP‐1, and SKOV3/shCHIP‐2 cells were analyzed by western blot with Snail‐, Slug‐, Vimentin‐, E‐cadherin‐, and CHIP‐specific antibodies and RT‐PCR with Snail‐ and CHIP‐specific primers. (B) CHIP‐dependent ubiquitylation of endogenous Snail. SKOV3/shCont, SKOV3/shCHIP‐1, and SKOV3/shCHIP‐2 cells were treated with 10 μm MG132 for 12 h before harvest. Lysates were immunoprecipitated using anti‐Snail antibody, followed by western blot with anti‐Ub antibody (upper). Whole‐cell lysates were immunoblotted with anti‐Snail (middle) and anti‐CHIP antibody (bottom) to demonstrate Snail and CHIP expression. Brackets indicate ubiquitylated Snail. (C) Increase of endogenous Snail protein stability by CHIP depletion. SKOV3/shCont and SKOV3/shCHIP‐1 cells were treated with 20 μg·mL^−1^ CHX for the indicated times before harvest, and western blot was performed with Snail‐ and CHIP‐specific antibodies. (D) Morphological changes of SKOV3 cells after inhibition of CHIP. SKOV3/shCont, SKOV3/shCHIP‐1, and SKOV3/shCHIP‐2 cells were visualized by phase‐contrast microscopy (upper) or confocal microscopy after staining with FITC‐conjugated phalloidin (lower). Scale bars, 40 μm (phalloidin images). (E) Migration of SKOV3 cells increased by inhibition of CHIP. SKOV3/shCont, SKOV3/shCHIP‐1, and SKOV3/shCHIP‐2 cells were analyzed by a wound‐healing assay visualizing wound closure by phase‐contrast microscopy. (F) Invasion of SKOV3 cells increased by inhibition of CHIP. SKOV3/shCont, SKOV3/shCHIP‐1, and SKOV3/shCHIP‐2 cells were seeded onto Matrigel matrix‐coated upper chambers, and fold changes of invaded cells were measured after 16 h. Data are mean ± SD of three individual, triplicate experiments. *P <* *0.01 as determined by paired Student's *t*‐test.

Snail is a central factor of EMT and directly contributes to repression of epithelial genes in mesenchymal cells. The ability of CHIP to downregulate Snail led us to investigate whether upregulation of Snail protein expression in CHIP‐depleted cells might repress epithelial markers and alter cellular phenotype. To find suitable models for testing the involvement of CHIP in Snail‐mediated EMT, we sought cell lines in which the Snail protein undergoes proteasomal degradation and found that in several cancer cell lines we tested, the expression levels of CHIP correlated negatively with the Snail expression levels (Fig. S3A), and MG132 treatment increased Snail protein levels in SW480, SW620, SKOV3, and OVCAR3 cells (Fig. S3B). Accordingly, we evaluated whether the depletion of CHIP could increase Snail expression, induce EMT, and enhance metastatic abilities in stable CHIP shRNA transfectants of these cell lines. However, CHIP depletion did not induce a decrease in E‐cadherin expression in SW480 cells, despite a marked increase in Snail expression (Fig. S4A). In SW620 cells, CHIP depletion even failed to increase Snail expression (Fig. S4B). However, we found that in SKOV3 cells, CHIP depletion led not only to an increase in Snail expression, but also to the downregulation of E‐cadherin and upregulation of Vimentin (Fig. 3A). When we rescued the expression of wild‐type CHIP or CHIP‐K30A, but not CHIP‐H260Q, in CHIP‐depleted SKOV3 (shCHIP‐1) cells, the expression of Snail was completely reduced to the same level as SKOV3 cells (Fig. S5). We also found that depletion of CHIP expression markedly reduced endogenous Snail ubiquitylation (Fig. 3B) and increased Snail protein stability in SKOV3 cells (Fig. 3C). Interestingly, the morphology of CHIP‐depleted SKOV3 (shCHIP‐1 and shCHIP‐2) cells was distinct from that of the control (shCont) cells; whereas the control cells remained tightly attached and exhibited typical epithelial cell characteristics, CHIP‐depleted SKOV3 cells were more diffuse and had lost cell–cell contact (Fig. 3D, upper). Confocal microscopy of phalloidin‐stained cells also confirmed the presence of filopodia, lamellipodia, and microspikes in CHIP‐depleted SKOV3 cells, whereas control cells exhibited less staining and no cellular protrusions (Fig. 3D, lower). These results indicate that CHIP depletion could induce EMT by enhancing Snail expression in SKOV3 cells.

Next, we investigated whether CHIP depletion could alter the migratory properties of SKOV3 cells by conducting a wound‐healing assay to determine the effect of CHIP knockdown on cell motility. After 16 h, we found that when compared with controls, an increased number of CHIP‐depleted SKOV3 cells had migrated into the scratch wound (Fig. 3E). Similarly, in an *in vitro* invasion assay, we detected a highly significant increase in the number of invasive CHIP‐depleted SKOV3 cells relative to controls (Fig. 3F). However, all cells exhibited similar growth rates under identical growth conditions (Fig. S6A), indicating that the increased migration and invasion observed in response to CHIP depletion were independent of proliferation rates. We also found that depletion of CHIP expression in OVCAR3, another ovarian cancer cell line, increased Snail expression, induced EMT phenotypes, and enhanced cancer cell migration and invasion ability (Fig. S7). In summary, CHIP depletion appears to increase ovarian cancer cell migration and invasion by inducing Snail‐mediated EMT.

### 3.4 Snail is essential for CHIP depletion‐induced EMT and increase of migration and invasion abilities in ovarian cancer cells

**Figure 4:**
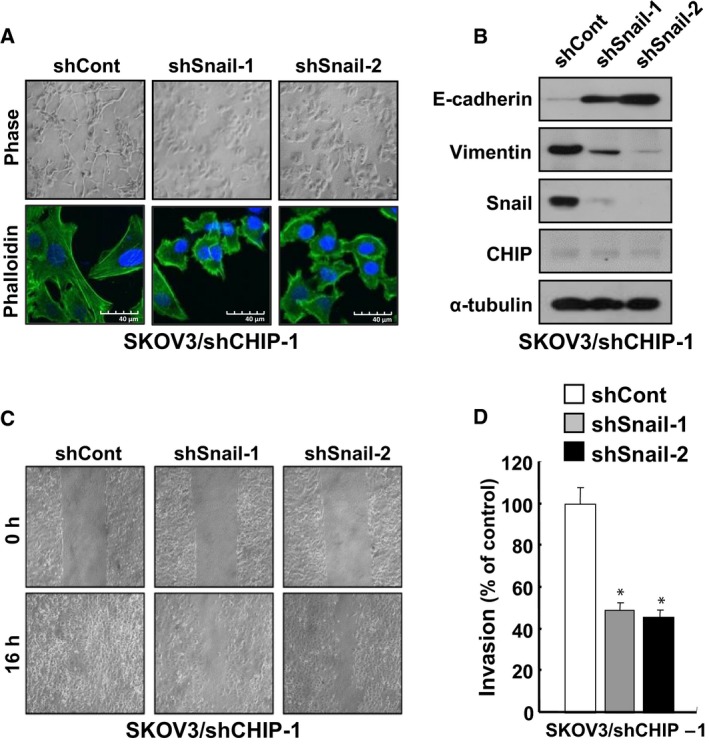
Snail is essential for CHIP depletion‐induced EMT and increase of migration and invasion abilities in ovarian cancer cells. (A) Morphological changes of SKOV3/shCHIP‐1 cells induced by Snail knockdown. SKOV3/shCHIP‐1/shCont, SKOV3/shCHIP‐1/shSnail‐1, and SKOV3/shCHIP‐1/shSnail‐2 cells were visualized by phase‐contrast microscopy (upper) or confocal microscopy after staining with FITC‐conjugated phalloidin (lower). Scale bars, 40 μm (phalloidin images). (B) Inhibition of Snail causes changes of EMT markers in CHIP‐depleted SKOV3 cells. Lysates of SKOV3/shCHIP‐1/shCont, SKOV3/shCHIP‐1/shSnail‐1, and SKOV3/shCHIP‐1/shSnail‐2 cells were analyzed by western blot with the indicated antibodies. (C) Inhibition of Snail decreases migration of CHIP‐depleted SKOV3 cells. SKOV3/shCHIP‐1/shCont, SKOV3/shCHIP‐1/shSnail‐1, and SKOV3/shCHIP‐1/shSnail‐2 cells were analyzed by a wound‐healing assay visualizing wound closure by phase‐contrast microscopy. (D) Inhibition of Snail decreases invasion of CHIP‐depleted SKOV3 cells. SKOV3/shCHIP‐1/shCont, SKOV3/shCHIP‐1/shSnail‐1, and SKOV3/shCHIP‐1/shSnail‐2 cells were seeded onto Matrigel matrix‐coated upper chambers, and fold changes of invaded cells were measured after 16 h. Data are mean ± SD of three individual, triplicate experiments. *P <* *0.01 as determined by paired Student's *t*‐test.

To investigate whether Snail is an important mediator of EMT in CHIP‐depleted SKOV3 cells, lentiviral shRNA (shSnail‐1 and shSnail‐2) was used to downregulate Snail expression in CHIP‐depleted SKOV3/shCHIP‐1 cells. Depletion of Snail expression led to a reversal of the EMT‐associated morphological changes induced by CHIP depletion; specifically, Snail and CHIP double‐depleted SKOV3 cells (shSnail‐1 and shSnail‐2) were tightly attached and exhibited typical epithelial cell characteristics with no cellular protrusions (Fig. 4A). In addition, Snail depletion reversed the repression of E‐cadherin and upregulation of Vimentin observed in CHIP‐depleted SKOV3 cells (Fig. 4B), suggesting that increased Snail expression is critical for the induction of EMT in CHIP‐depleted SKOV3 cells.

In a subsequent evaluation to determine whether Snail depletion could alter the migratory and invasive properties of CHIP‐depleted SKOV3 cells, we found that Snail depletion considerably suppressed both the migratory abilities (Fig. 4C) and invasiveness of these cells (Fig. 4D). Again, we confirmed that cellular growth rates were not significantly different (Fig. S6B), indicating that decreased tumor cell migration and invasion in response to Snail knockdown were not associated with proliferation. These results suggest that Snail upregulation plays a key role in CHIP depletion‐induced SKOV3 cell migration and invasion.

### 3.5 Snail is essential for CHIP depletion‐induced peritoneal dissemination and lung metastasis *in vivo*

**Figure 5:**
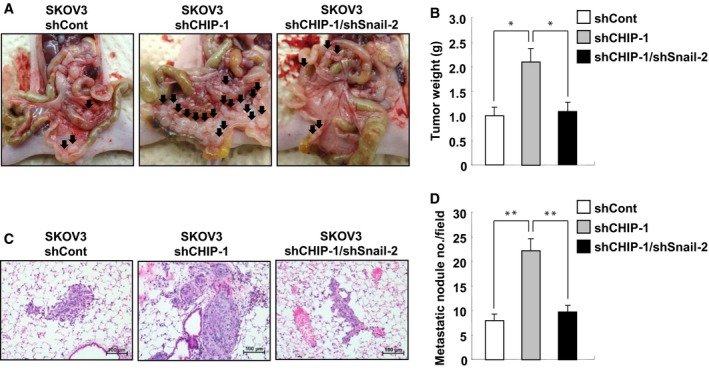
Snail is essential for CHIP depletion‐induced peritoneal dissemination and lung metastasis *in vivo*. (A) *In vivo* roles of CHIP and Snail in the peritoneal dissemination of SKOV3 cells. SKOV3/shCont, SKOV3/shCHIP‐1, and SKOV3/shCHIP‐1/shSnail‐2 cells (2 × 10^6^) were suspended in 200 μL PBS and intraperitoneally injected into BALB/c mice (15 mice/group). After 6 weeks, tumor burden and ascite formation were estimated. Arrows indicate disseminated tumors. (B) At autopsy, tumors were excised and weighed. Data are mean ± SD of 15 mice/group. *P <* *0.05 as determined by paired Student's *t*‐test. (C) *In vivo* roles of CHIP and Snail in lung metastasis of SKOV3 cells. Sections of lung organs excised from mice described in (A) were stained with hematoxylin and eosin. Representative images from one of fifteen mice per group. Scale bars, 100 μm. (D) Number of metastatic lung nodules per field. The number of metastatic lung nodules in individual mice was counted under the microscope. Data are mean ± SD of 15 mice/group. **P <* *0.01 as determined by paired Student's *t*‐test.

Ovarian cancers metastasize primarily on the peritoneum and rarely migrate to distant sites. The resulting peritoneal implants are characterized by the adhesion and invasion of tumor cells into the peritoneum, leading to miliary dissemination (Lengyel, 2010). Therefore, to analyze the *in vivo* roles of CHIP and Snail in the peritoneal dissemination of ovarian cancer cells, female BALB/c nude mice were injected intraperitoneally with control SKOV3 (shCont), CHIP‐depleted SKOV3 (shCHIP‐1), or Snail and CHIP double‐depleted SKOV3 (shCHIP‐1/shSnail‐2) cells, after which tumors were allowed to grow for 6 weeks. After 6 weeks, the peritoneal surface tumor burden was significantly higher (*P *<* *0.05) in mice that had received SKOV3/shCHIP‐1 cells (2.09 ± 0.33 g) vs. control cells (1.01 ± 0.22 g), whereas Snail knockdown suppressed the effect of CHIP knockdown (1.08 ± 0.24 g; Fig. 5A,B). Furthermore, significant lung metastasis was observed in mice that had received SKOV3/shCHIP‐1 cells vs. those that had received with control cells; again Snail knockdown suppressed this effect (Fig. 5C,D, Fig. S8). Taken together, these data indicate that CHIP depletion could enhance both peritoneal dissemination and lung metastasis of ovarian cancer cells *in vivo* in a Snail‐dependent manner.

### 3.6 Inverse correlation between Snail and CHIP expression in primary ovarian tumor tissues

**Figure 6:**
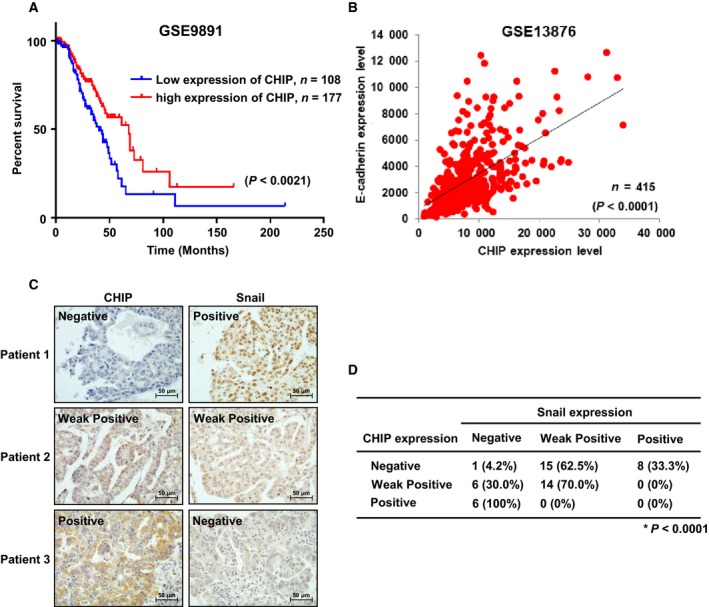
Inverse correlation between Snail and CHIP expression in primary ovarian tumor tissues. (A) Correlation between CHIP expression and overall survival in 285 ovarian cancer patients through analysis of a public microarray data set (GSE9891). A Kaplan–Meier plot analysis showed overall survival depending on the expression level of CHIP. *P* values were calculated using a log‐rank test. (B) Comparison of CHIP expression level with E‐cadherin expression level in 415 ovarian tumor samples using a microarray data set (GSE13876). (C) Representative immunohistochemical staining of human ovarian tumor tissues with CHIP or Snail antibody. Scale bars, 50 μm. (D) Inverse correlation between CHIP and Snail protein expression in primary ovarian cancer tissues. The expression patterns of CHIP and Snail in 50 ovarian cancer samples were determined by immunohistochemistry and are summarized. The correlation between CHIP and Snail was analyzed using Fisher's exact test (*P *<* *0.0001).

To address the clinical significance of CHIP in ovarian cancer patients, we first analyzed public microarray data sets (GSE9891 and GSE13876; Crijns *et al*., 2009; Tothill *et al*., 2008) in 285 and 415 ovarian tumor samples, respectively, and found that abundance of CHIP is related to good prognosis (Fig. 6A) and CHIP expression levels are positively correlated with E‐cadherin expression (Fig. 6B). Finally, to verify the correlation between CHIP and Snail protein expression levels in clinical samples, we conducted a tissue microarray analysis of 50 ovarian cancer tissue specimens and observed a strong negative correlation between CHIP and nuclear Snail expression (Fig. 6C,D). Tumors with strong CHIP expression exhibited little or no nuclear Snail expression. In contrast, tumors lacking CHIP expression exhibited strong nuclear Snail expression. Our findings indicate that Snail expression in ovarian cancer patients can be negatively regulated by CHIP.

## 4 Discussion

**Figure 7:**
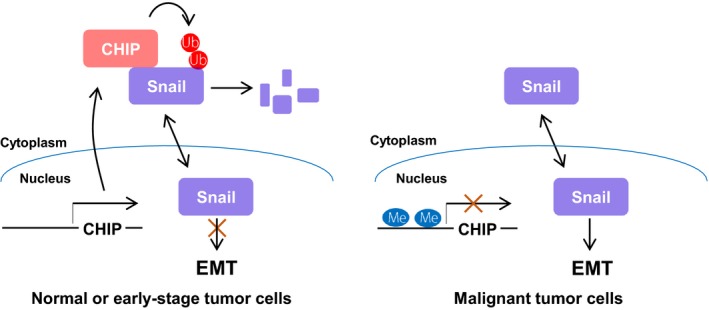
Proposed model for CHIP‐mediated ubiquitylation and degradation of Snail in ovarian tumor cells. In normal or early‐stage tumor cells, CHIP is highly expressed and effectively degrades Snail through ubiquitylation, contributing to reduced EMT (left). In malignant tumor cells, CHIP expression is repressed by promoter hypermethylation, and increased nuclear Snail protein induces EMT (right).

In this study, we revealed a new metastasis mechanism in which downregulation of CHIP expression resulted in accumulation of Snail, which acted as an inducer of EMT process, thereby contributing to the metastasis of ovarian cancer cells by enabling migration and invasion. Importantly, our study together with clinical evidence demonstrated that CHIP may acts as a metastasis‐suppressive E3 ubiquitin ligase which is downregulated in malignant ovarian cancer patients. Consistent with our findings, a previous report demonstrated that CHIP expression is significantly decreased in colorectal tumor tissues and downregulation of CHIP is correlated with CHIP promoter hypermethylation (Wang *et al*., 2014). Besides CHIP, many tumor‐suppressive E3 ubiquitin ligase genes have been known to be downregulated by methylation of the promoter regions frequently in the development of human malignancies (James *et al*., 1994; Mizuno *et al*., 2002; Zhang *et al*., 2007). Using MethHC (http://methhc.mbc.nctu.edu.tw), a database of DNA methylation and gene expression in human cancers that systematically integrates a large collection of DNA methylation and mRNA expression profile data (Huang *et al*., 2015), we confirmed that CHIP promoter hypermethylation was present in most human malignant tumor samples (Fig. S9). Although further studies are needed to completely elucidate the correlation between CHIP promoter methylation and Snail expression in ovarian cancer, our data suggest that CHIP is strongly expressed in normal or early‐stage ovarian tumor cells, where it effectively degrades Snail through ubiquitylation, thus suppressing EMT. In contrast, CHIP expression is repressed in malignant tumor cells, and the resulting high levels of Snail expression could promote EMT (Fig. 7).

We also demonstrated that CHIP can ubiquitylate and degrade Snail in a GSK‐3β‐independent manner by showing CHIP ubiquitylates and degrades Snail‐6SA mutant (Fig. S2). But, there is a confliction in the localization of CHIP and Snail‐6SA mutant. CHIP is localized only in the cytoplasm (Fig. 1D), and Snail‐6SA mutant could be in the nucleus because Snail‐6SA mutant could not be exported to the cytoplasm due to the lack of GSK‐3β phosphorylation sites. However, there are two possibilities to interact Snail‐6SA mutant with CHIP in the cytoplasm. First, Snail can interact with CHIP in the cytoplasm before entering into the nucleus. Second, while phosphorylation by GSK‐3β is abolished in Snail‐6SA mutant, we cannot exclude the possibility that Snail‐6SA may be phosphorylated by another kinase, such as PKD1, and could be exported into the cytoplasm by GSK‐3β‐independent manner. PKD1 has been known to phosphorylate Snail at Serine‐11 and induce nuclear export of Snail (Du *et al*., 2010). However, CHIP might ubiquitylate and degrade Snail in PKD1‐independent manner because we have shown that Snail‐ΔSNAG retains the ability to associate with CHIP (Fig. 1F). But, all of these results cannot completely rule out the possibility that the ubiquitylation of Snail by CHIP is phosphorylation‐independent. In particular, since the SRD of Snail is important for its interaction with CHIP (Fig. 1F), it is possible that phosphorylation of Snail is prerequisite for interaction with CHIP. We are now trying to find molecule that plays important role for Snail's cytoplasmic localization and its interaction with CHIP.

In this study, to find suitable models for testing the involvement of CHIP in Snail‐mediated EMT, we made many CHIP‐depleted stable cell lines. However, depletion of CHIP expression did not induce a decrease in E‐cadherin expression in SW480 cells, despite a marked increase in Snail expression (Fig. S4A). The zinc finger domain of Snail has been shown to play a role in nuclear localization, which is mediated by Pak1 phosphorylation on Ser246 in the zinc finger (Yang *et al*., 2005). Pak1 phosphorylation of Snail could support Snail's accumulation in the nucleus as well as its repressor functions. Although the levels of Pak1 activity in SW480 cells should be checked, the relatively low levels of Pak1 activity may explain why increase of Snail expression could not induce a decrease in E‐cadherin expression in SW480 cells. In SW620 cells, there might be other E3 ubiquitin ligases that regulate subcellular levels of Snail, besides CHIP, which may explain why CHIP depletion even failed to increase Snail expression (Fig. S4B). We also found that Snail did not interact with CHIP in SW620 cells (data not shown).

In summary, we report here for the first time, to the best of our knowledge, CHIP as an inducer of Snail ubiquitylation and degradation. We showed that CHIP controls the stability of Snail and Snail‐mediated EMT, migration, invasion, and *in vivo* metastatic potential of ovarian cancer cells. Although further studies are needed to completely elucidate the correlation between CHIP promoter methylation and Snail expression in ovarian cancer, we concluded from our study that CHIP may help modulate Snail and improve our understanding of the biology of ovarian cancer metastasis.

## 5 Conclusion

In conclusion, this study identified CHIP as a new E3 ubiquitin ligase that interacts with Snail to induce ubiquitin‐mediated proteasomal degradation. Depletion of CHIP expression can induce EMT and enhance metastatic potential of ovarian cancer cells in Snail‐dependent manner. Moreover, we found that abundance of CHIP is related to good prognosis and expression of CHIP is negatively correlated with Snail expression in ovarian cancer patients. Our study provided new insight into the post‐translational regulation mechanism of Snail during the development of ovarian cancer metastasis.

## Conflict of interest

The authors declare no conflict of interest.

## Author contributions

S‐MP, S‐HP, K‐JR, I‐KK, HS, and JY designed the research. S‐MP, S‐HP, K‐JR, and I‐KK performed the major portion of the experiments. HH, H‐JK, S‐HK, K‐SH, HK, and MK performed parts of the research. BIC, JDH, and NHK performed the animal experiments and analyzed immunohistochemical staining results. EMH, J‐YP, JIY, HJC, CH, and KDK supervised experiments and discussed and interpreted results of the study. S‐MP, S‐HP, and JY wrote the manuscript. JY supervised the project. All authors read and approved the final manuscript.

## Supporting information


**Fig. S1.** CHIP ubiquitylates Snail under denaturing conditions.
**Fig. S2.** CHIP ubiquitylates Snail in a GSK‐3β‐independent manner.
**Fig. S3.** Negative correlation of the CHIP expression level with Snail expression level in several cancer cell lines.
**Fig. S4.** Depletion of CHIP expression cannot induce EMT in SW480 and SW620 cells.
**Fig. S5.** Rescue of wild‐type CHIP or CHIP‐K30A expression can reduce the expression of Snail in CHIP‐depleted SKOV3/shCHIP‐1 cells.
**Fig. S6.** Effect of CHIP and Snail depletion on the proliferation of SKOV3 cells.
**Fig. S7.** Depletion of CHIP expression induces EMT and increases the migration and invasion abilities of OVCAR3 cells.
**Fig. S8.** Representative images of hematoxylin and eosin‐stained entire lung section from mice described in Fig. 5C.
**Fig. S9.** Methylation of the CHIP promoter in cancer tissue samples analyzed using the MethHC (http://MethHC.mbc.nctu.edu.tw) database.
**Table S1.** shRNA sequences used in this study.
