# Supplementary material for: Downregulation of CHIP promotes ovarian cancer metastasis by inducing Snail‐mediated epithelial–mesenchymal transition
Source: Mol Oncol. 2019 Apr 8;13(5):1280–95. doi: 10.1002/1878-0261.12485 (PMC6487736; doi:10.1002/1878-0261.12485)
Supplement: Supplementary file 1 — Fig. S1. CHIP ubiquitylates Snail under denaturing conditions. Fig. S2. CHIP ubiquitylates Snail in a GSK‐3β‐independent manner. Fig. S3. Negative correlation of the CHIP expression level with Snail expression level in several cancer cell lines. Fig. S4. Depletion of CHIP expression cannot induce EMT in SW480 and SW620 cells. Fig. S5. Rescue of wild‐type CHIP or CHIP‐K30A expression can reduce the expression of Snail in CHIP‐depleted SKOV3/shCHIP‐1 cells. Fig. S6. Effect of CHIP and Snail depletion on the proliferation of SKOV3 cells. Fig. S7. Depletion of CHIP expression induces EMT and increases the migration and invasion abilities of OVCAR3 cells. Fig. S8. Representative images of hematoxylin and eosin‐stained entire lung section from mice described in Fig. 5C. Fig. S9. Methylation of the CHIP promoter in cancer tissue samples analyzed using the MethHC (http://MethHC.mbc.nctu.edu.tw) database. Table S1. shRNA sequences used in this study. [file MOL2-13-1280-s001.pdf]

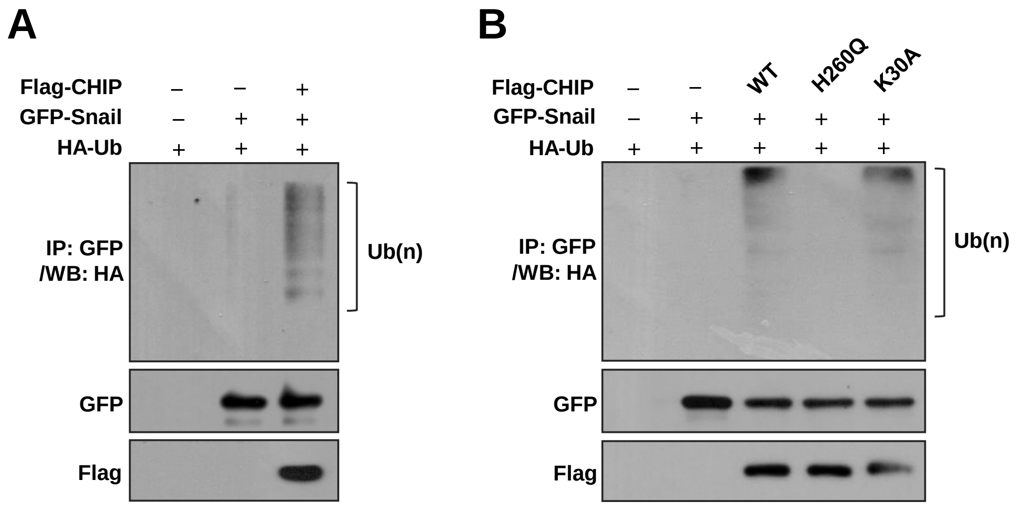

Fig. S1. CHIP ubiquitylates Snail under denatured condition. (A) CHIP-dependent ubiquitylation of Snail in denatured condition. HEK293T cells were transfected with expression plasmids encoding GFP-Snail, Flag-CHIP, and HA-ubiquitin and treated with 10 μM MG132 for 12 hr before harvest. Cells were lysed with denaturing lysis buffer and the denatured lysates were immunoprecipitated using anti-GFP antibody, followed by western blot with anti-HA antibody (upper). Whole cell lysates were immunoblotted with anti-GFP (middle) and anti-Flag antibody (bottom) to demonstrate Snail and CHIP expression. Brackets indicate ubiquitylated Snail. (B) Ubiquitylation of Snail by CHIP WT, H260Q, and K30A mutants in denatured condition. Ubiquitylation assays using HEK293T cells transfected with the indicated plasmids were performed as in (A).

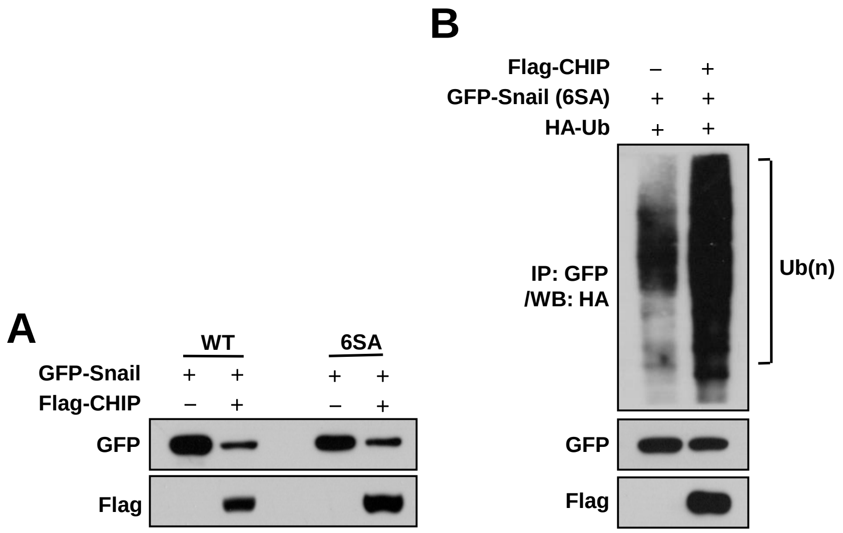

Fig. S2. CHIP ubiquitylates Snail in a GSK-3β-independent manner. (A) Degradation of GSK-3β-non-phosphorylation mutant of Snail by CHIP. Putative GSK-3β phosphorylation sites of Snail were mutated to arginine in the Snail 6SA mutant. HEK293T cells were transfected with GFP-Snail WT and Flag-CHIP or GFP-Snail 6SA and Flag-CHIP, respectively. Western blot analysis was performed using anti-GFP and anti-Flag antibodies. (B) Ubiquitylation of GSK-3β-non-phosphorylation mutant of Snail by CHIP. HEK293T cells were transfected with GFP-Snail 6SA, Flag-CHIP, and HA-ubiquitin and treated with MG132 (10 μM) for 12 hr before harvest. Lysates were immunoprecipitated using anti-GFP antibody, followed by western blot with anti-HA antibody (upper). Whole cell lysates were immunoblotted with anti-GFP (middle) and anti-Flag antibody (bottom) to demonstrate Snail and CHIP expression. Brackets indicate ubiquitylated Snail.

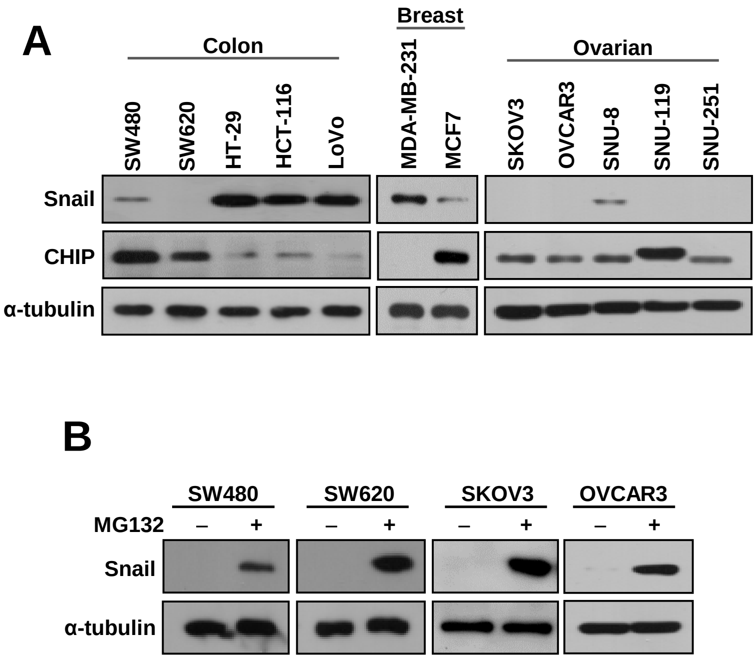

Fig. S3. Negative correlation of the CHIP expression level with Snail expression level in several cancer cell lines. (A) Immunoblot analysis in several cancer cell lines with antibodies against Snail or CHIP. (B) Increase of Snail stability by MG132 in SW480, SW620, SKOV3, and OVCAR3 cells. Cells were treated with MG132 (10 μM) for 6 hr and analyzed by western blot with anti-Snail antibody.

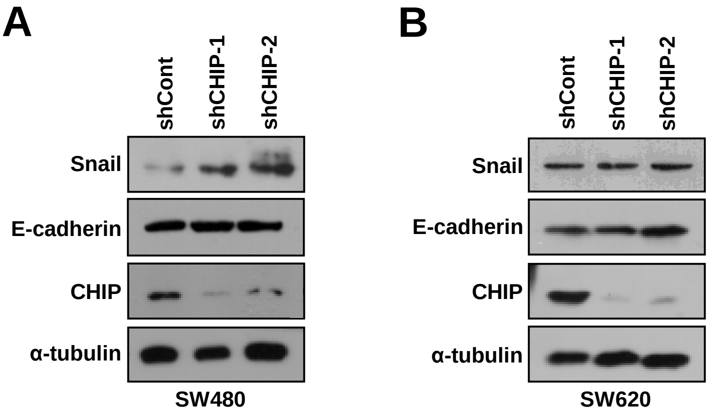

Fig. S4. Depletion of CHIP expression cannot induce EMT in SW480 and SW620 cells. SW480 (A) and SW620 (B) cells were infected with lentiviral shCHIP. Western blot analysis was performed using CHIP-, Snail-, and E-cadherin-specific antibodies.

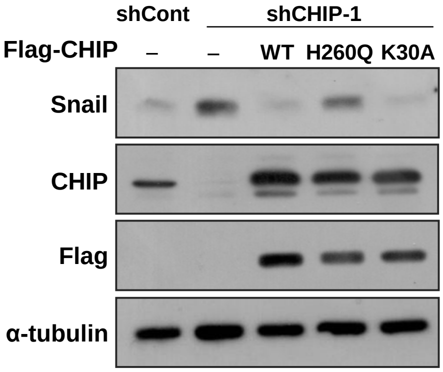

Fig. S5. Rescue of wild-type CHIP or CHIP-K30A expression can reduce the expression of Snail in CHIP-depleted SKOV3/shCHIP-1 cells. Lysates of SKOV3/shCHIP-1 cells transfected with indicated plasmids were analyzed by western blot with Snail-, CHIP-, and Flag-specific antibodies.

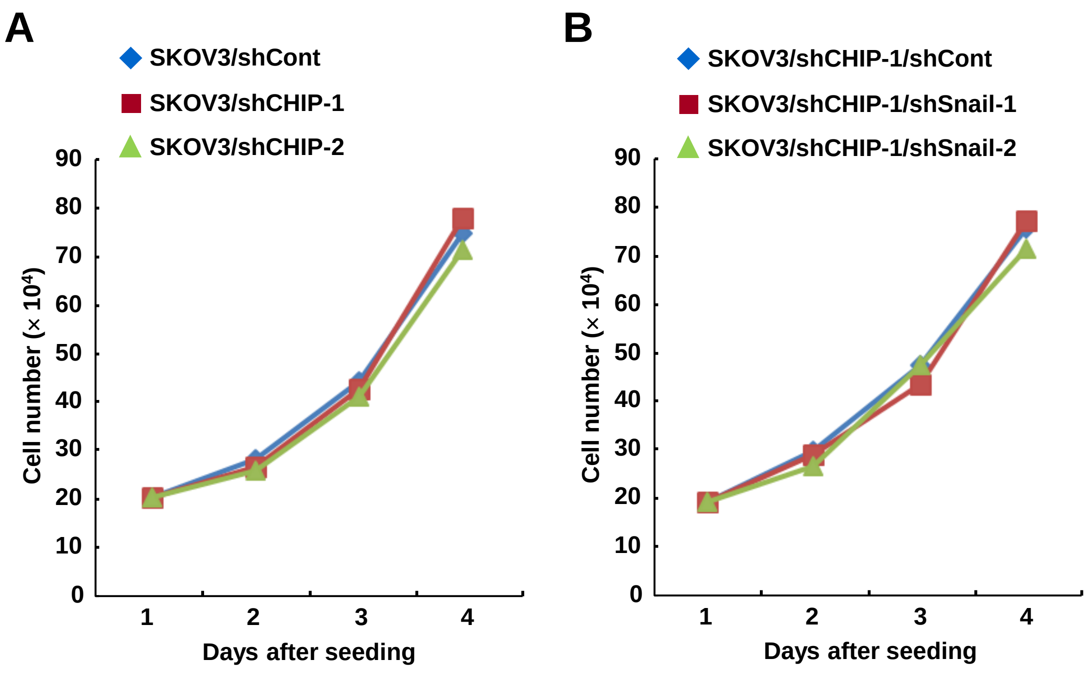

Fig. S6. Effect of CHIP and Snail depletion on the proliferation of SKOV3 cells. The indicated cells were placed in a 6-well plate at a concentration of 2 X 10^4 cells per well. After incubation for 1 to 4 days, the viable cells were counted with a hemocytometer after trypan blue staining. We repeated the experiments in (A) and (B) three times, each in triplicate.

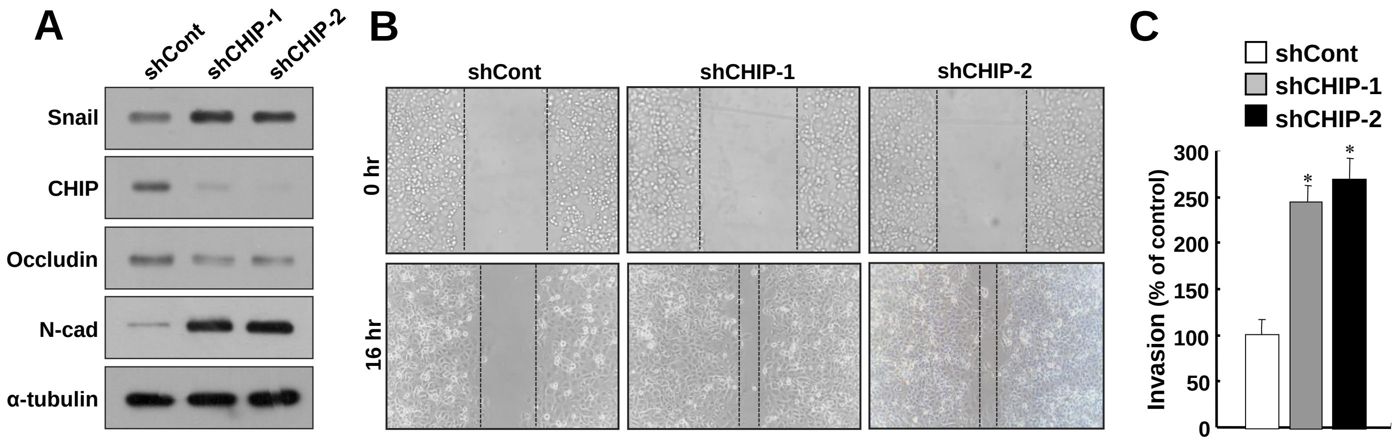

Fig. S7. Depletion of CHIP expression induces EMT and increases the migration and invasion abilities of OVCAR3 cells. (A) Inhibition of CHIP induces changes of EMT markers in OVCAR3 cells. Lysates of OVCAR3/shCont, OVCAR3/shCHIP-1, and OVCAR3/shCHIP-2 cells were analyzed by western blot with Snail-, Occludin-, N-cadherin-, and CHIP-specific antibodies (B) Migration of OVCAR3 cells increased by inhibition of CHIP. OVCAR3/shCont, OVCAR3/shCHIP-1, and OVCAR3/shCHIP-2 cells were analyzed by a wound-healing assay visualizing wound closure by phase-contrast microscopy. (C) Invasion of OVCAR3 cells increased by inhibition of CHIP. OVCAR3/shCont, OVCAR3/shCHIP-1, and OVCAR3/shCHIP-2 cells were seeded onto Matrigel matrix-coated upper chambers, and fold changes of invaded cells were measured after 16 hr. Data are mean ± SD of three individual, triplicate experiments. *, $p < 0.01$ as detrmined by paired Student's *t*-test.

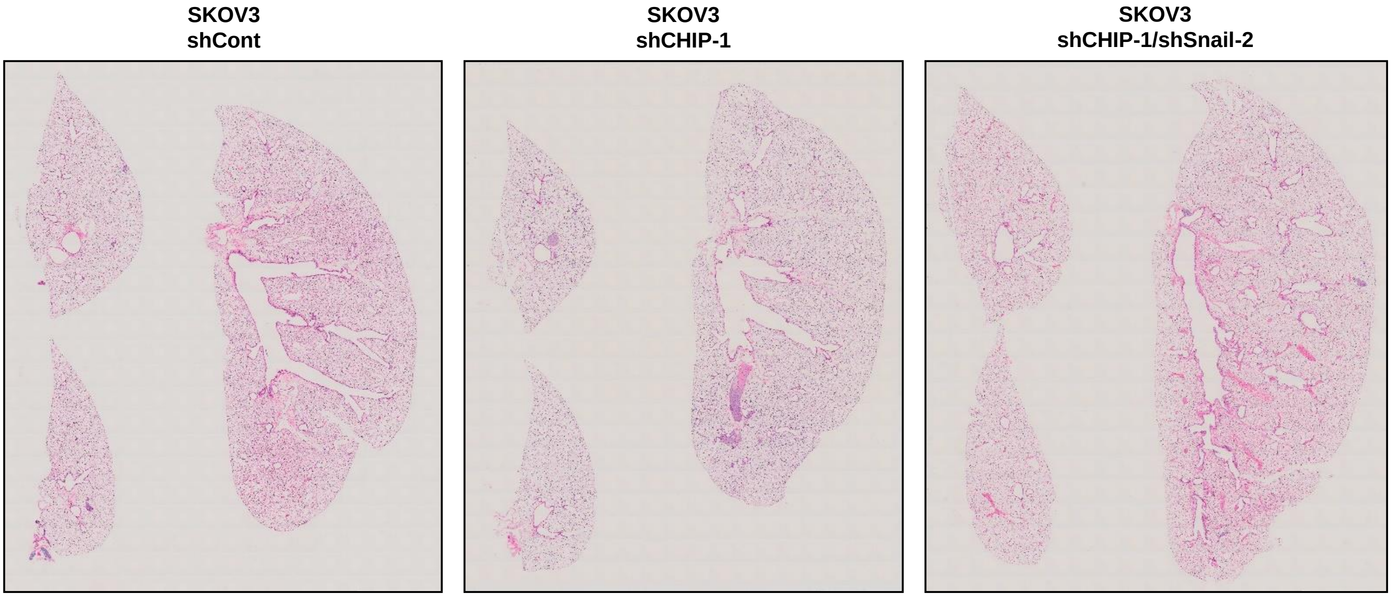

Fig. S8. Representative images of hematoxylin and eosin-stained entire lung section from mice described in Fig. 5C. Each lung section was scanned by using BX61VS (Olympus).

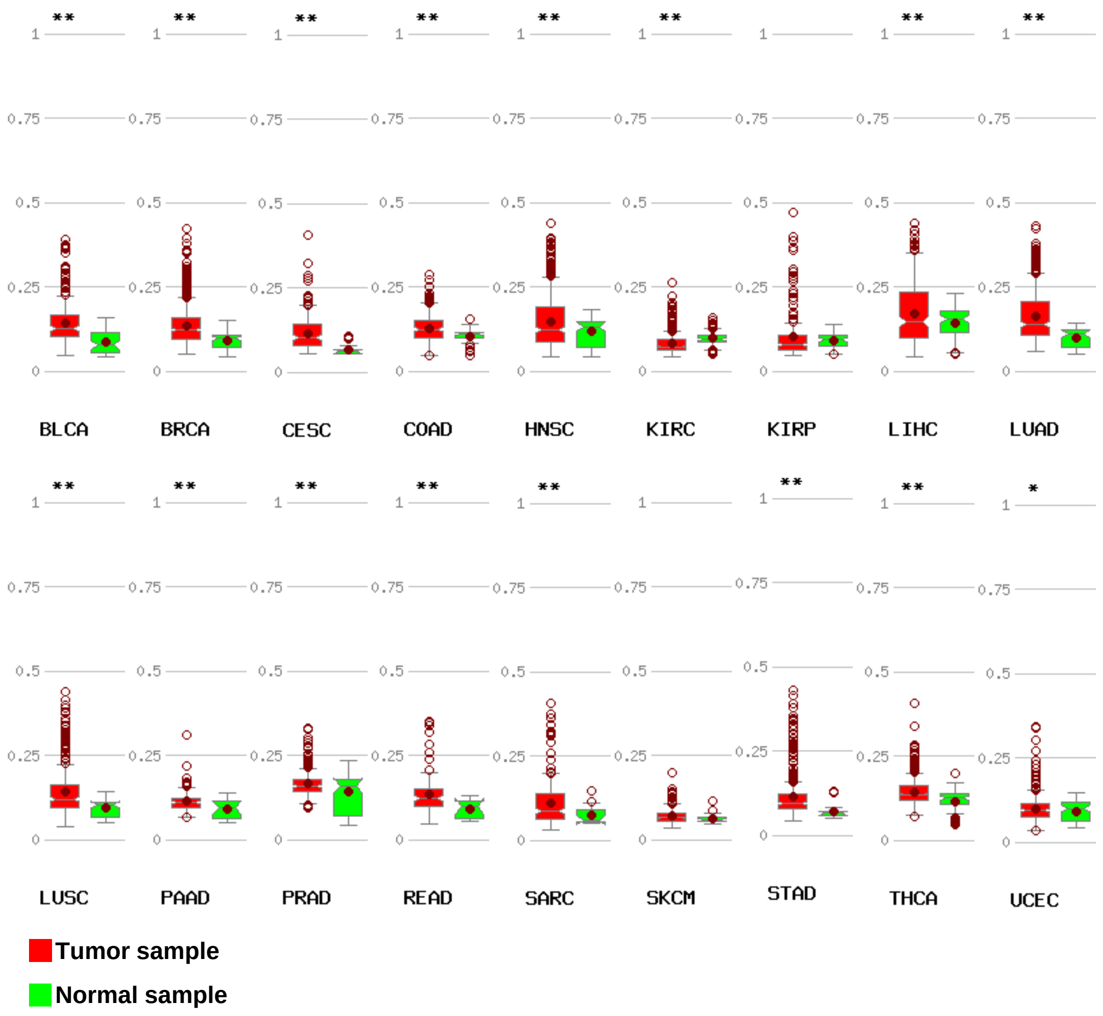

Fig. S9. Methylation of the CHIP promoter in cancer tissue samples analyzed using the MethHC (http://MethHC.mbc.nctu.edu.tw) database. BLCA: Bladder Urothelial Carcinoma, BRCA: Breast Invasive Carcinoma, CESC: Cervical Squamous Cell Carcinoma and Endocervical Adenocarcinoma, COAD: Colon Adenocarcinoma, HNSC: Head and Neck Squamous Cell Carcinoma, KIRC: Kidney Renal Clear Cell Carcinoma, KIRP: Kidney Renal Papillary Cell Carcinoma, LIHC: Liver Hepatocellular Carcinoma, LUAD: Lung Adenocarcinoma, LUSC: Lung Squamous Cell Carcinoma, PAAD: Pancreatic Adenocarcinoma, PRAD: Prostate Adenocarcinoma, READ: Rectum Adenocarcinoma, SARC: Sarcoma, SKCM: Skin Cutaneous Melanoma, STAD: Stomach Adenocarcinoma, THCA: Thyroid Carcinoma, UCEC: Uterine Corpus Endometrial Carcinoma. *, $p < 0.05$; **, $p < 0.005$.

Table S1. shRNA sequences used in this study

Name of Gene	shRNA sequences
CHIP	
shCHIP-1	5’-CCGGCCCAAGTTCTGCTGTTGGACTCTCGAGAGTCCAACAGCAGAACTTGGGTTTTT-3’
shCHIP-2	5’-CCGGGAAGAGGAAGAAGCGAGACATCTCGAGATGTCTCGCTTCTTCCTCTTCTTTTT-3’
Snail	
shSnail-1	5’-CCGGCCACTCAGATGTCAAGAAGTACTCGAGTACTTCTTGACATCTGAGTGGTTTTTG-3’
shSnail-2	5’-CCGGCCAAGGATCTCCAGGCTCGAACTCGAGTTCGAGCCTGGAGATCCTTGGTTTTTG-3’
